# A Synthetic Curcuminoid Analogue, 2,6-Bis-4-(Hydroxyl-3-Methoxybenzylidine)-Cyclohexanone (BHMC) Ameliorates Acute Airway Inflammation of Allergic Asthma in Ovalbumin-Sensitized Mice

**DOI:** 10.1155/2021/9725903

**Published:** 2021-04-03

**Authors:** Chau Ling Tham, Sin Yee Yeoh, Chun Hao Ong, Hanis Hazeera Harith, Daud Ahmad Israf

**Affiliations:** Department of Biomedical Science, Faculty of Medicine and Health Sciences, Universiti Putra Malaysia, Serdang 43300, Malaysia

## Abstract

2,6-Bis-(4-hydroxyl-3-methoxybenzylidine) cyclohexanone (BHMC), a synthetic curcuminoid analogue, has been shown to exhibit anti-inflammatory properties in cellular models of inflammation and improve the survival of mice from lethal sepsis. We further evaluated the therapeutic effect of BHMC on acute airway inflammation in a mouse model of allergic asthma. Mice were sensitized and challenged with ovalbumin (OVA), followed by intraperitoneal administration of 0.1, 1, and 10 mg/kg of BHMC. Bronchoalveolar lavage fluid, blood, and lung samples were collected, and the respiratory function was measured. OVA sensitization and challenge increased airway hyperresponsiveness (AHR) and pulmonary inflammation. All three doses of BHMC (0.1-10 mg/kg) significantly reduced the number of eosinophils, lymphocytes, macrophages, and neutrophils, as well as the levels of Th2 cytokines (IL-4, IL-5 and IL-13) in bronchoalveolar lavage fluid (BALF) as compared to OVA­challenged mice. However, serum level of IgE was not affected. All three doses of BHMC (0.1-10 mg/kg) were effective in suppressing the infiltration of inflammatory cells at the peribronchial and perivascular regions, with the greatest effect observed at 1 mg/kg which was comparable to dexamethasone. Goblet cell hyperplasia was inhibited by 1 and 10 mg/kg of BHMC, while the lowest dose (0.1 mg/kg) had no significant inhibitory effect. These findings demonstrate that BHMC, a synthetic nonsteroidal small molecule, ameliorates acute airway inflammation associated with allergic asthma, primarily by suppressing the release of inflammatory mediators and goblet cell hyperplasia to a lesser extent in acute airway inflammation of allergic asthma.

## 1. Introduction

Allergic asthma is an inflammatory disease of the airways which is characterized by the infiltration of immune cells, including eosinophils, mast cells, lymphocytes, and neutrophils; activation of Th 2 cells that produce IL-4, IL-5, and IL-13 within the airway wall; mucus hypersecretion by goblet cells; and increased airway hyperresponsiveness (AHR) [1, 2]. Although most asthmatic patients respond well to currently available therapy (inhaled corticosteroids and *β*-agonists), the efficacy of such treatment is limited in some patients with severe asthma. This is thought to be associated with structural alterations known as airway remodeling (features include goblet cell hyperplasia and airway smooth muscle hypertrophy/hyperplasia), which could not be reversed by current treatments [3]. This ongoing problem has driven research into developing a more effective and specific therapy for asthma.

Apart from bronchodilators, research has been focusing on targeting the inflammatory process involved in the progression of asthma. Some targets include IgE, IL-4, IL-5, and IL-13, all of which are key players that drive or contribute to airway inflammation, remodeling, and hyperresponsiveness [4]. Another approach is to target the molecules upstream of these inflammatory events which regulate the expression of genes that commonly drive inflammatory reactions such as the transcription factor nuclear factor-kappa B (NF-*κ*B) or the p38 mitogen-activated protein kinase. Inhibitors used for this purpose are known as broad-spectrum noncorticosteroid anti-inflammatory treatments which may be useful in asthmatic cases where corticosteroids are poorly effective [4].

**Figure 1:**
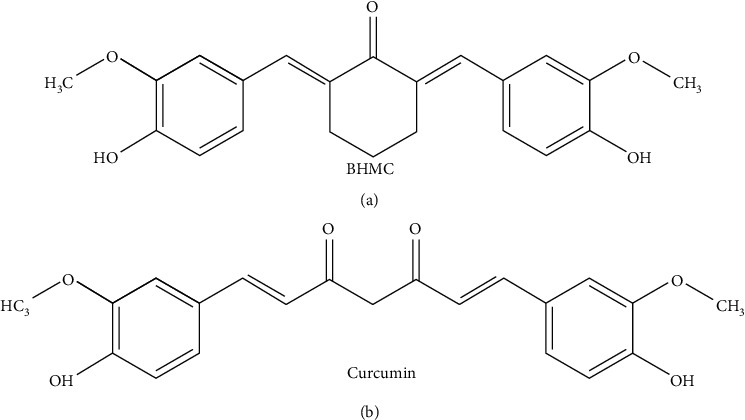
The chemical structure of (a) BHMC [2,6-bis-(4-hydroxyl-3-methoxybenzylidine)cyclohexanone] and (b) curcumin.

Curcumin (1,7-bis(4-hydroxy-3-methoxyphenyl)hepta-1,6-diene-3,5-dione) (Figure 1(b)) is an active compound isolated from the rhizomes of a plant called turmeric (*Curcuma longa* L. Zingiberaceae family) [5]. A range of biological and pharmacological activities have been described for curcumin including anticancer, anti-inflammatory, and antioxidant activity [6]. However, curcumin has not been approved clinically due to its poor bioavailability [7]. Therefore, there is a growing research interest in the potential of synthetic analogues of curcumin.

We had previously synthesized a structural derivative of curcumin known as 2,6-bis-(4-hydroxyl-3-methoxybenzylidine) cyclohexanone (BHMC) (Figure 1(a)) [8], which retains the phenolic OH group attributed to the anti-inflammatory activity of curcumin, with improved bioavailability compared to curcumin. The anti-inflammatory properties of BHMC has been validated in several cellular models of inflammation, which include RAW 264.7 murine macrophages, U937 human monoblastoid leukemic cells, and human umbilical vein endothelial cells (HUVECs) [9–11]. BHMC also exerts anti-invasive effect in human breast cancer cells as demonstrated by the reduction in invadopodia formation [12]. These findings suggest that BHMC possesses similar anti-inflammatory and antimetastatic properties compared to curcumin. At present, the anti-inflammatory potential of BHMC is only supported by in vitro evidence. Here, we report that BHMC is able to ameliorate allergic airway inflammation by suppressing the accumulation of inflammatory cells in the airways and bronchoalveolar lavage fluid (BALF), as well as the production of Th2 cytokines in BALF.

## 2. Materials and Methods

### 2.1. Materials

Sodium chloride, curcumin, methacholine, PBS tablet, glycerol, sodium phosphate dibasic, potassium phosphate monobasic, dexamethasone (DEX), OVA, aluminum hydroxide (ALUM), Tween-20, Tert-amyl alcohol, Wright stain, paraffin wax, 2,2,2-tribromoethanol, periodic acid, Alcian Blue, Schiff reagent, DPX mountant, and eosin and hematoxylin solution were purchased from Sigma Chemical Co. (St. Louis, MO, USA). Formalin, hydrochloric acid, acetic acid, methanol, and ethanol were purchased from Merck (NJ, USA). BD PrecisionGlide Needle 22G, BD OptEIA Mouse IL4, IL-5, IL-13, and IgE ELISA sets were purchased from BD Biosciences Pharmingen (San Diego, CA, USA).

### 2.2. Methods

#### 2.2.1. Experimental Animals

Six-to-eight-week-old male BALB/c mice were obtained from Animal Experimentation Unit, Faculty of Medicine and Health Sciences, Universiti Putra Malaysia, and allowed to acclimatize for 1 week prior to experiment. The experimental mice were housed in a temperature and humidity-controlled room with food and water provided *ad libitum* and were kept under a 12 hrs light/dark diurnal cycle. The experiments described below were performed in accordance with the current guidelines for the care of laboratory animals and the ethical guidelines for animal experimentation Animal Care and Use Committee (ACUC). This work has been approved by the Animal Experimentation Ethics Committee, Faculty of Medicine and Health Sciences, Universiti Putra Malaysia (UPM/FPSK/PADS/BR-UUH/00373).

#### 2.2.2. Immunization and Challenge

This study involved a mouse model of acute allergic asthma where OVA is used as the inducer. For OVA-induced acute airway inflammation of allergic asthma, immunization and challenge of experimental mice were performed as described previously [13]. Mice were randomly divided into 6 groups of 8 animals each. Each mouse was immunized intraperitoneally on days 0, 7, and 14 with 50 *μ*g chicken egg white ovalbumin (OVA; 500 *μ*g/mL) emulsified in ALUM (10% *w*/*v*) in a total volume of 100 *μ*L of PBS (pH 6.5). From day 21 to day 23, mice were consecutively challenged with OVA (1% *w*/*v* in PBS, pH 7.4) by inhalation using the OMRON ultrasonic nebulizer NE-U17 (Kyoto, Japan) for 30 min. A group of sensitized mice were challenged with PBS instead of 1% OVA (designated as the PBS group).

#### 2.2.3. Treatment

Three different doses of BHMC (0.1, 1.0, 10 mg/kg) were selected based on a previous study [10]. BHMC was dissolved in 90% distilled water with 5% ethanol and 5% Tween-20. One hour prior to 1% OVA challenge, the experimental mice were intraperitoneally administered with either the vehicle (designated as the OVA group), BHMC (0.1, 1.0, 10 mg/kg), or 3 mg/kg of dexamethasone (designated as the DEX group).

#### 2.2.4. Determination of Airway Hyperresponsiveness (AHR)

AHR was induced as described previously [14]. Briefly, each mouse was exposed to incremental doses of methacholine (6.25, 12.5, 25, and 50 mg/mL) for 3 min each, 24 hrs after the last OVA challenge. This procedure was performed in an enclosed chamber, and the readings were measured and recorded for 5 min after each nebulization using Buxco Whole Body Plethysmometer System (New Brighton, MN, USA). This system evaluates the Enhanced Pause (Penh) index of AHR to indicate changes in airway resistance, which is a function of total pulmonary airflow during the respiratory cycle. Penh correlates with pulmonary airflow resistance or obstruction. It is expressed as Pause x PEP/PIP or (expiration time/relaxation time-1) x (peak expiratory pressure/peak inspiratory pause).

#### 2.2.5. Bronchoalveolar Lavage Fluid (BALF) Collection and Leukocytes Count

BALF collection from asthmatic mice and the subsequent leukocytes count were performed as described previously [13]. The mice were sacrificed by administering a lethal dose of avertin (500 mg/kg) on day 27. BALF was collected by tracheal cannulation with a 22G feeding needle, followed by flushing of the lung lobes with ice cold PBS (0.9 mL; pH 7.2) for four times. Precautions were taken to ensure that the lungs were inflated during the flushing, and there was no leakage of lavage fluid from the trachea. The BALF collected were then centrifuged at 200xg, 4°C for 10 min, and the supernatant was stored at -80°C for further analysis. The pellet was resuspended in 500 *μ*L of PBS and cytosmears were prepared on a Hettich centrifuge with cytospin adaptors. Smears were air-dried overnight and stained with Wright's stain. The stained slides were air-dried and then viewed under the Leica microscope for differential cell count analysis (Allendale, NJ, USA). Total cell count was performed by mixing the remaining cell suspension with an equal volume of trypan blue and counted using a hemocytometer.

#### 2.2.6. Histopathological Studies

Histopathological studies were performed as described previously [13]. Following BALF collection, 10% formalin was administered into the lungs and trachea of the mice using a 22G feeding needle. The lung was then removed and fixed in 10% formalin for at least 72 hrs. The tissue was dehydrated and paraffin-embedded using Leica Automated Tissue Processor TP 1020 (Leica Instrument Gmb, Wetzlar, Germany). The embedded tissue was cut into 4 *μ*m sections for histological examination. Tissue sections were subjected to hematoxylin and eosin (H&E) staining to quantify airway inflammation by obtaining the average number of inflammatory cells (the total number of inflammatory cells in peribronchial and perivascular regions divided by the total number of airways/blood vessels per section). The tissue sections were stained with Periodic Acid Schiff (PAS) to quantify goblet cell hyperplasia by obtaining the average number of goblet cells (total number of PAS-positive cells in the airways divided by total number of airways per section). Three sections were counted for each animal. Histological analysis was carried out in a blinded fashion by two investigators in the laboratory.

#### 2.2.7. ELISA

Blood was collected immediately after euthanization through cardiac puncture using a 26G needle. The concentration of total IgE in serum and the concentrations of IL-4, IL-5, and IL-13 in the supernatants of BALF was determined by ELISA according to the manufacturer's protocol (BD Biosciences Pharmingen, San Diego, CA, USA). The absorbance was measured at 450 nm with a microplate reader (UVM 340, ASYS Hitech GmbH, Austria, Europe). The levels of IgE and cytokines in the samples were interpolated from their respective standard curves.

#### 2.2.8. Statistical Analysis

The results were analyzed by one-way ANOVA (comparing the treatment groups against the OVA group), using the SPSS version 17.0 software. The changes in the OVA group was also statistically analyzed against the PBS group. Post hoc comparisons were carried out using Dunnett's test to determine the most effective dose. *P* values of less than 0.05 were considered to be significant.

## 3. Results

### 3.1. Effect of BHMC on Airway Hyperresponsiveness (AHR)

**Figure 2:**
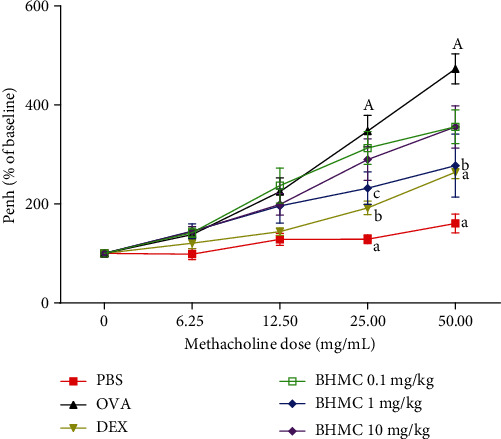
The effect of BHMC on airway hyperresponsiveness in response to methacholine. BALB/c mice were sensitized and challenged with OVA 1 hr after treatment with DEX (3 mg/kg) or indicated doses of BHMC (*n* = 8 per group). 24 hrs after the last OVA challenge, the mice were exposed to incremental doses of methacholine (6.25, 12.5, 25, and 50 mg/mL) in an enclosed chamber for 3 min, and the readings were recorded for 5 min after each nebulization. The values are expressed as mean ± SEM. c: *P* < 0.05, b: *P* < 0.01, and a: *P* < 0.005, significantly different from the OVA group. A: *P* < 0.005, significantly different from the PBS group.

As shown in Figure 2, the OVA group showed a significantly increased percentage of Penh following the administration of 25 and 50 mg/mL methacholine, in comparison with the PBS group. Notably, mice receiving dexamethasone or 1 mg/kg BHMC had significantly reduced percentage of Penh compared to the OVA group at both 25 mg/mL (*P* < 0.05) and 50 mg/mL (*P* < 0.01) of methacholine. In contrast, no significant change was observed in mice receiving 0.1 or 10 mg/kg BHMC (*P* > 0.05). These results indicate that intraperitoneal administration of 1 mg/kg BHMC was able to reduce bronchoconstriction in OVA-sensitized and OVA-challenged mice.

### 3.2. Effect of BHMC on Total Leukocytes and Differential Cell Count

**Figure 3:**
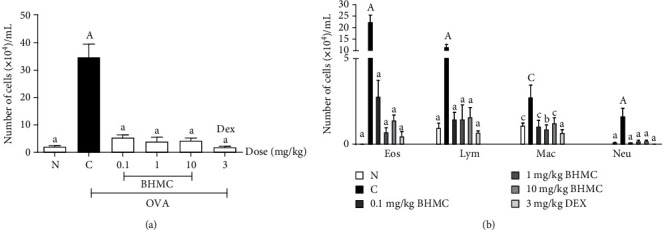
The effects of BHMC on the (a) total leukocytes and (b) differential cell counts in the BALF from OVA-induced mice. The BALF was collected from euthanized mice (*n* = 8 per group), and the cells were stained with Wright stain. The numbers of leukocytes, eosinophils, neutrophils, lymphocytes, and macrophages were counted under the microscope. The values are expressed as mean ± SEM. c: *P* < 0.05, b: *P* < 0.01, and a: *P* < 0.005, significantly different from the OVA group. C: *P* < 0.05 and A: *P* < 0.005, significantly different from the N group. N represents the PBS group whereas C represents the OVA-challenged group.

The effect of BHMC in this murine model of OVA-induced airway inflammation was further assessed by determining the numbers of leukocytes, eosinophils, neutrophils, macrophages, and lymphocytes in the BALF collected. All doses of BHMC tested caused a marked reduction (*P* < 0.005) in the total number of leukocytes (Figure 3(a)), as well as individual numbers of eosinophils, neutrophils, lymphocytes, and macrophages (Figure 3(b)), even at the lowest dose of 0.1 mg/kg. These findings suggest that BHMC at a concentration ranging between 0.1 and 10 mg/kg was able to attenuate the numbers of leukocytes found in BALF, including eosinophils, neutrophils, lymphocytes, and macrophages, all of which are critical inflammatory cells in asthma. The effect of BHMC observed was comparable to the DEX group.

### 3.3. Effect of BHMC on the Infiltration of Inflammatory Cells

**Figure 4:**
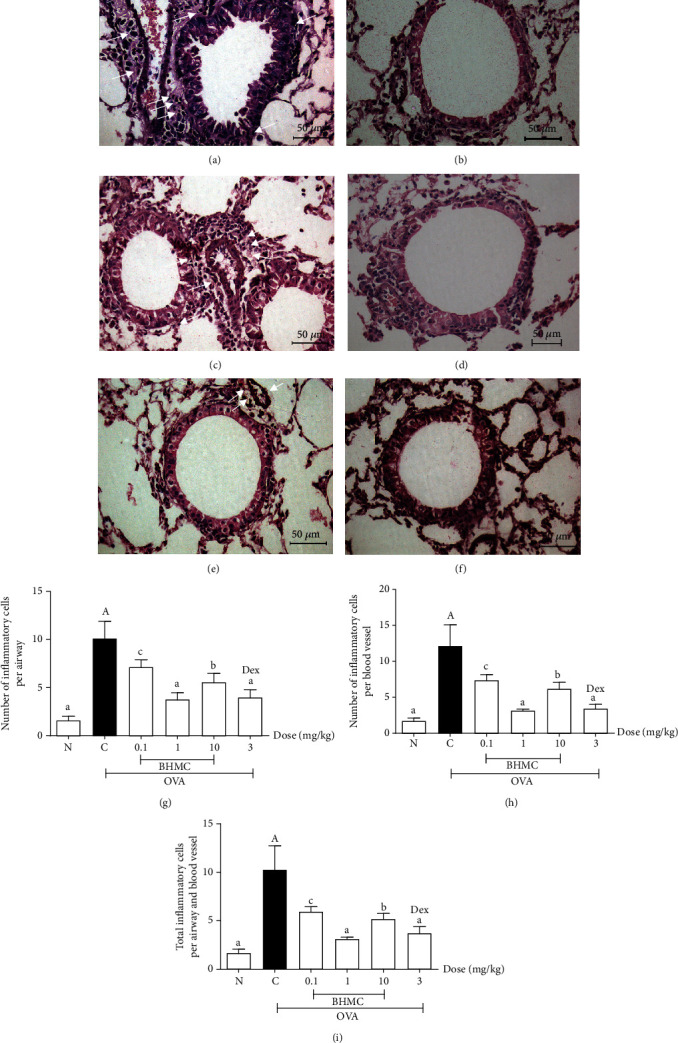
The effects of BHMC on the infiltration of inflammatory cells in peribronchial and perivascular regions of OVA-induced mice. After BALF was collected, the lungs and trachea of the mice (*n* = 8 per group) were processed into tissue sections and stained with H&E for the evaluation of cellular infiltration. The representative images of lung sections from the (a) OVA group, (b) PBS group, (c) 0.1 mg/kg BHMC group, (d) 1 mg/kg BHMC group, (e) 10 mg/kg BHMC group, and (f) DEX group. The inflammatory cells are indicated by the white arrows. Representative images of lung sections were taken at 400x magnification (40x for objective lens and 10x for ocular lens) using a light microscope. The results are also expressed quantitatively by counting the number of infiltrated cells at the (g) peribronchial region, (h) perivascular region, and (i) total for both peribronchial and perivascular regions. The values are expressed as mean ± SEM. c: *P* < 0.05, b: *P* < 0.01, and a: *P* < 0.005, significantly different from the OVA group. A: *P* < 0.005, significantly different from the N group.

The effect of BHMC on the infiltration of inflammatory cells in the lung tissue sections was also examined. The OVA group exhibited extensive infiltration of inflammatory cells into the airways, as indicated by increased density and number of inflammatory cells around the airways (peribronchial) or the blood vessels (perivascular) (Figure 4(a)) compared to the PBS group (Figure 4(b)). Treatment with BHMC (0.1, 1, and 10 mg/kg) (Figures 4(c)–4(e)) or dexamethasone (Figure 4(f)) significantly inhibited the infiltration of inflammatory cells surrounding the airways (peribronchial) and blood vessels (perivascular).

In order to examine the effect of BHMC on peribronchial and perivascular inflammation quantitatively, the number of inflammatory cells surrounding each airway and blood vessel was counted. Figures 4(g) and 4(h) show that all doses of BHMC (0.1, 1, and 10 mg/kg) significantly inhibited the infiltration of inflammatory cells surrounding the airways (peribronchial) and blood vessels (perivascular). This finding was further confirmed by the overall inhibitory effect of BHMC on lung inflammation, obtained by combining the average of infiltrated inflammatory cells of both the perivascular and peribronchial regions (Figure 4(i)). Among all the doses tested, the inhibitory effect of 1 mg/kg BHMC was comparable to that of dexamethasone (*P* < 0.001). These results show that BHMC could suppress the infiltration of inflammatory cells in the lungs of OVA-sensitized and OVA-challenged mice.

### 3.4. Effect of BHMC on Goblet Cells Hyperplasia

**Figure 5:**
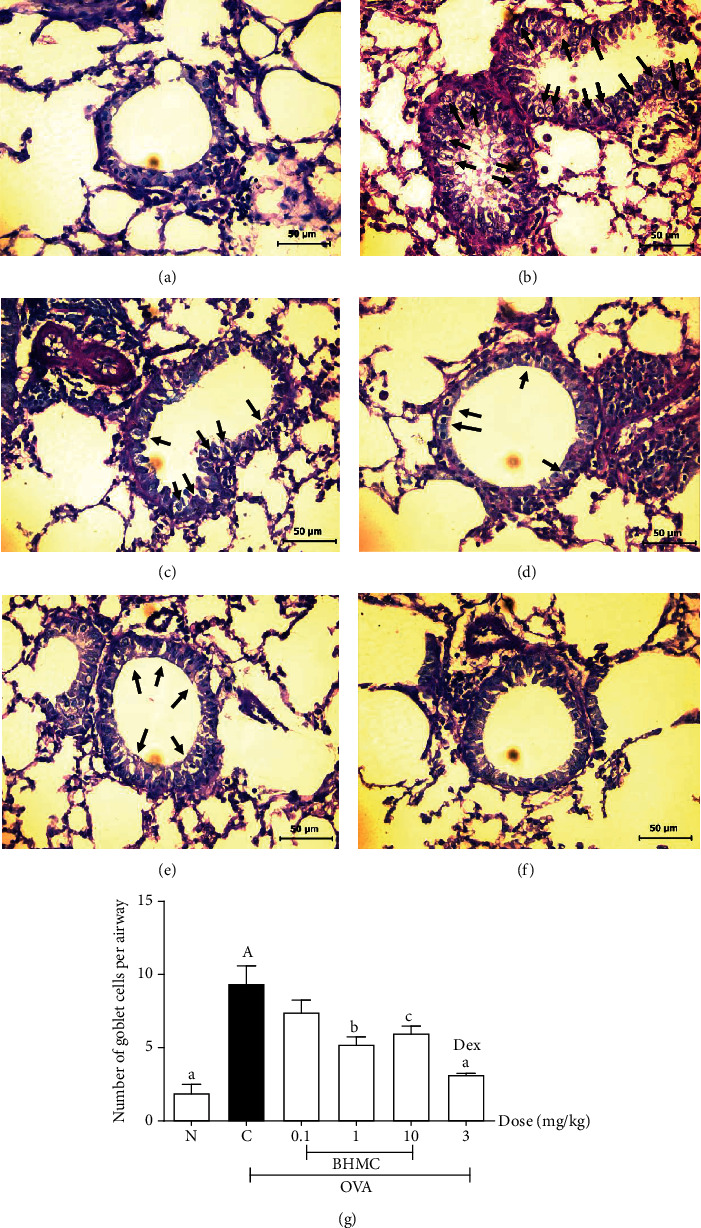
The effect of BHMC on goblet cell hyperplasia in OVA-induced mice. After the BALF was collected, the lungs and trachea of the mice were processed into tissue sections (*n* = 8 per group) and stained with periodic acid Schiff (PAS) stain for histopathological evaluation of goblet cell hyperplasia. The representative images of lung sections from the (a) PBS group, (b) OVA group, (c) 0.1 mg/kg BHMC group, (d) 1 mg/kg BHMC group, (e) 10 mg/kg BHMC group, and (f) DEX group. The goblet cells are indicated by black arrows. The representative images of lung sections were taken at 400x magnification (40x for objective lens and 10x for ocular lens) using a light microscope. (g) The number of goblet cells in each airway was counted, and the sum of the goblet cells was divided by the total number of airways in each slide. The values are expressed as mean ± SEM. b: *P* < 0.01 and a: *P* < 0.005, significantly different from the OVA group. A: *P* < 0.005, significantly different from the N group.

Airway goblet cell hyperplasia is a characteristic feature of airway remodeling in the OVA-induced asthmatic mouse model [15]. As shown in Figure 5, goblet cell hyperplasia was clearly observed in the bronchial airways of OVA-challenged mice (Figure 5(b)) compared to the PBS group (Figure 5(a)). Notably, treatment with dexamethasone (Figure 5(f)) resulted in markedly reduced mucus production and goblet cell hyperplasia (*P* < 0.005). Compared to the DEX group, mice receiving 1 and 10 mg/kg BHMC also significantly reduced goblet cell hyperplasia in OVA-challenged mice (*P* < 0.01 and *P* < 0.05, respectively), but to a lesser extent (Figure 5(g)). Such an effect was not observed at 0.1 mg/kg BHMC.

### 3.5. Effects of BHMC on the Levels of IL-4, IL-5, IL-13, and IgE

**Figure 6:**
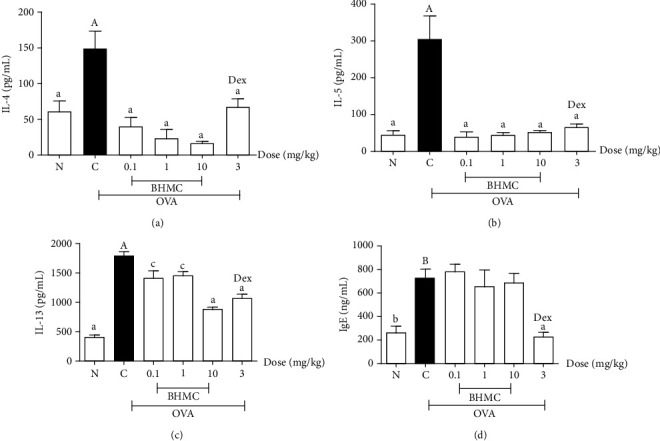
The effects of BHMC on the levels of IL-4, IL-5, and IL-13 in BALF and serum IgE from OVA-induced acute asthmatic mice. The BALF was collected from euthanized mice (*n* = 8 per group) and the levels of (a) IL-4, (b) IL-5, and (c) IL-13 were measured using ELISA kits. (d) The levels of IgE in serum was also measured by ELISA according to the manufacturer's protocol. The values are expressed as mean ± SEM performed in triplicates. c: *P* < 0.05 and a: *P* < 0.005, significantly different from the OVA group. B: *P* < 0.01 and A: *P* < 0.005, significantly different from the N group.

The production of Th2-cytokines and IgE plays an important role in promoting allergic airway inflammation and hyperresponsiveness in asthma [16]. Therefore, the effects of BHMC treatment on the levels of IL-4, IL-5, and IL-13 in BALF, and serum IgE levels in OVA-induced asthmatic mouse model were determined using ELISA. As expected, the OVA group exhibited significantly elevated levels of IL-4 (Figure 6(a)), IL-5 (Figure 6(b)), IL-13 (Figure 6(c)), and IgE (Figure 6(d)) compared to the PBS group. These OVA-induced Th2 cytokines and IgE production were significantly suppressed by dexamethasone (*P* < 0.005). In general, all three doses of BHMC significantly attenuated the levels of IL-4, IL-5, and IL-13 in BALF of OVA-challenged mice. Of note, all three doses of BHMC exhibited a comparable inhibitory effect on IL-4 and IL-5 levels in BALF to dexamethasone. In contrast, the inhibitory effect of BHMC on IL-13 secretion was only comparable to that of dexamethasone at 10 mg/kg. While BHMC was able to inhibit the production of Th2 cytokines in BALF obtained from OVA-challenged mice, no change was observed in serum IgE levels (*P* > 0.05). These results indicate that BHMC could suppress the production of Th2 cytokines in OVA-challenged mice, independent of IgE production.

## 4. Discussion

Inflammation, bronchoconstriction, and overproduction of mucous which obstruct the airways are processes that contribute to the narrowing of the airways in asthma [17]. In this study, the anti-inflammatory properties of BHMC in the context of allergic airway inflammation were investigated by assessing three parameters, namely, the inflammatory cell counts and the levels of Th2 cytokines in BALF, as well as the infiltration of inflammatory cells in the peribronchial or perivascular regions of lung tissue. This study demonstrates that BHMC strongly attenuated the number of total leukocytes, eosinophils, neutrophils, lymphocytes, and the levels of Th2 cytokines (IL-4, IL-5, and IL-13) in BALF obtained from OVA-challenged mice. These changes were correlated with greatly reduced accumulation of inflammatory cells around the airways (peribronchial) and blood vessels (perivascular) at all BHMC doses tested. The increased number of peribronchial and perivascular inflammatory cell infiltrates contributes to peribronchial or perivascular lung inflammation in OVA-challenged mice. These observations are in agreement with a previous study which highlighted that both peribronchial and perivascular inflammation are the integral components of lung immune response in acute and chronic lung inflammation induced by OVA [18].

In a type 2 inflammatory pathway, antigen-presenting cells will cause Th2 differentiation, which in turn secretes cytokines like IL-4, IL-5, and IL-13. The cytokines IL-4 and IL-13 facilitate class switching of B cells to synthesize IgE, while IL-5 stimulates the activation of eosinophils [19]. This study provides evidence that BHMC not only has a potent inhibitory effect on the infiltration of inflammatory cells in the lungs of OVA-challenged mice but also able to suppress the production of Th2 cytokines in the BALF. Notably, BHMC showed a weaker effect on IL-13 compared to IL-4 and IL-5. This observation suggests that BHMC may interfere with specific factors that induce IL-4 and IL-5 gene expression but not IL-13. This may be possible given that previous studies have demonstrated that these Th2 cytokines can be regulated differentially by certain factors. For example, one study showed that IFN-*α* induction could downregulate IL-13 but not IL-4 [20]. Another study reported that a transient activation of the transcription factor NFAT is sufficient to turn on the expression of IL-13, whereas IL-4 and IL-5 genes require prolonged NFAT activation [21]. BHMC could be interfering with the activation of NFAT, resulting in stronger reduction of IL-4 and IL-5 compared to IL-13. Nonetheless, the mechanisms by which BHMC attenuates the production of Th2 cytokines remain to be identified.

Despite its strong anti-inflammatory effect, BHMC did not alter the serum IgE levels. The role of IgE in the development of airway inflammation is well-established. The Fc region of IgE antibodies can bind to mast cells which can be activated upon allergen binding to the bound IgE. This results in the degranulation of mast cells and the release of inflammatory mediators such as histamine, leukotriene, and prostaglandin [16]. Our findings suggest that BHMC exerts its anti-inflammatory effect mainly through its inhibitory effect on IL-4 and IL-5 expression. Among the Th2 cytokines, IL-13 has been shown to significantly enhance the synthesis of IgE with suboptimal level of IL-4 [22]. As such, the inability of BHMC to strongly inhibit IL-13 expression may explain the lack of changes in IgE levels in mice treated with BHMC.

As the main source of mucin which gives mucus the gel-like consistency, goblet cell hyperplasia underlies excessive production of mucus in the progression of asthma [2]. This study also demonstrates that BHMC is able to significantly attenuate AHR and goblet cell hyperplasia but only at 1 mg/kg. These results suggest that BHMC has limited ability to suppress AHR and goblet cell hyperplasia following an OVA challenge. It is likely that this resulted from the limited inhibitory effect of BHMC on IL-13 expression, the cytokine that induces AHR and goblet cell hyperplasia. The role of IL-13 in promoting AHR and goblet cell hyperplasia is evident from a previous report which demonstrated that mice receiving IL-13 developed eosinophilic inflammation, AHR, and goblet cell hyperplasia compared to control mice. In the same study, dexamethasone was unable to inhibit AHR and goblet cell hyperplasia despite a strong inhibitory effect on eotaxin expression and eosinophilic inflammation [23]. Based on our findings, BHMC exerts a significant anti-inflammatory activity but has limited effect on AHR and goblet cell hyperplasia, similar to the activity of dexamethasone described previously by Kibe et al. (2003). Furthermore, these findings also reflect that infiltration of inflammatory cells within the airways in OVA-challenged mice may not have a causal relationship with AHR or goblet cell hyperplasia due to the fact that suppression of inflammation by BHMC did not cause effective reduction in AHR and goblet cell hyperplasia. This suggests that AHR and goblet cell hyperplasia can persist independent of inflammation.

Three different doses of BHMC (0.1, 1, and 10 mg/kg) were used throughout the experiments to examine if the compound manifests a dose-dependent trend in the results. However, the results show no obvious dose-dependent relationship between the doses and the effects of BHMC. Instead of the highest dose (10 mg/kg), the intermediate dose (1 mg/kg) of BHMC exhibited the strongest inhibitory effect on AHR, goblet cell hyperplasia, and inflammatory cell count in peribronchial and perivascular regions. Based on these observations, we proposed that BHMC could be exerting a hormetic-like biphasic effect, such that the optimal effect was observed at the intermediate dose rather than the highest dose of treatment. This finding is similar to a previous study from our research group which investigated the effect of BHMC (0.5, 1, 2, and 10 mg/kg) on CLP-induced lethal sepsis [10]. We reported a similar hormetic-like biphasic trend where 1 mg/kg BHMC offered the best protection from septic death when compared to the lower dose (0.5 mg/kg) and higher doses (2 and 10 mg/kg). The U-shaped dose response of BHMC may occur as a result of modest overcompensation in response to a disruption in homeostasis. The exact reason of the hormetic effects exhibited by BHMC is not yet understood since hormetic responses could be influenced by a combination of mechanisms which may vary among different cell types [24]. Further studies will have to be conducted to elucidate this mechanism.

Although the link between hyperresponsiveness of the airway smooth muscle and airway inflammation is quite established, the precise mechanisms that link airway inflammation and AHR remain unclear [25]. In this study, we demonstrate that BHMC exerts a stronger suppressive effect on the accumulation of inflammatory cells especially eosinophils in BALF, the infiltration of inflammatory cells in the lung as well as the level of Th2 cytokines such as IL-4 and IL-5, in comparison to AHR and goblet cell hyperplasia. As goblet cell hyperplasia is a feature of airway remodelling in asthma and remodelling contributes to AHR, this work suggests that BHMC primarily acts on inflammation rather than airway remodeling in acute airway inflammation of allergic asthma.

Although this study is unable to directly compare the activity of BHMC with curcumin due to the lack of curcumin treatment group, curcumin has been demonstrated to attenuate both airway inflammation and airway remodeling based on its ability to reduce goblet cell hyperplasia, mucus hypersecretion, and thickening of airway wall [26]. Furthermore, while BHMC is unable to reduce the IgE levels in the present study despite its strong anti-inflammatory activity, other studies have reported that intranasal administration of curcumin could significantly suppress serum IgE levels with a comparable effect to dexamethasone [26, 27]. Taken together, these findings suggest that curcumin's activity vary with BHMC, a derivative of curcumin, to some extent and the potential application of BHMC may be limited to eosinophilic inflammatory conditions. Nonetheless, further studies to investigate the exact mechanism of action of BHMC on the regulation of Th2 cytokines such as gene expression and pathway analysis are required in order to explain its mild inhibition on IL-13 expression and its inability to suppress IgE production. Apart from the lack of curcumin treatment group, another limitation of this study is that the OVA-sensitized mouse model applied does not truly reflect the conditions in corticosteroid-resistant allergic asthma. As the ultimate objective of this study is to develop BHMC as the alternative treatment for severe asthma cases that do not respond well to corticosteroid treatment, further studies are necessary to assess the effectiveness of BHMC in a corticosteroid refractory model. The role of BHMC in the oxidative stress responses, which play important role in the pathophysiology of asthma and hyperresponsiveness, will also be further investigated in future studies. Our previous study had demonstrated that BHMC acts as a potent inhibitor of nitric oxide [9]. As nitric oxide reacts with superoxide radicals (O2•–) to form peroxynitrite (ONOO-) which is a powerful oxidant that can contribute to airway inflammation via activation of NF-*κ*B signaling [28], it is believed that suppression of nitric oxide synthesis by BHMC would help to reduce oxidative stress and result in suppression of NF-*κ*B-mediated proinflammatory cytokines production. Nonetheless, further studies should be conducted to delineate the exact role of BHMC in the oxidative stress responses of airway inflammation.

## 5. Conclusion

In summary, BHMC has the potential to ameliorate acute airway inflammation of allergic asthma in OVA-sensitized mice. BHMC treatment suppressed the number of leukocytes and the levels of Th2 cytokines in BALF. The infiltration of inflammatory cells in peribronchial and perivascular regions was also effectively reduced by BHMC treatment. Nevertheless, BHMC did not show significant inhibitory effect on the levels of IgE.

## Data Availability

The data that support the findings of this study are available on request from the corresponding author.
